# Experiments in Reference to Section II. of a Paper in the “May No. of the Review,” on “the Perceptive Power of the Spinal Cord”

**Published:** 1858-07

**Authors:** George R. Paton

**Affiliations:** Galt, Canada West


ART. III.—*Experiments in Reference to Section II. of a Paper in the "May Number of the Review," entitled "The Perceptive Power of the Spinal Cord."* By GEORGE R. PATON, M.D., of Galt, Canada West.

*Experiment* 4, April 26th, 1858.—I divided the spinal cord of a frog immediately behind the skull, and passing an instrument into the encephalon, removed every portion of nervous matter anterior to the division of the cord. Respiration ceased.

I then placed the frog in a vessel containing a little water, submerging it till about two-thirds of its body were covered, but not allowing the water to come in contact with the divided portion of the spine; then permitting it to remain quiescent for a considerable time, till the effects of the operation had subsided, I applied irritation, and observed the phenomena.

I touched with the point of a needle the integuments of its right lumbar region, and it raised up its right hind foot and passed its toes over the part. I irritated in a similar manner the left lumbar region, and it passed the toes of its left hind foot over the part. I irritated the integuments of its right shoulder, and it raised up its right hind leg and passed its toes over the part; on irritating the integuments of its left shoulder, a similar movement was performed with its left hind foot. I then touched with the point of a needle the integuments of its right fore leg, and it withdrew it. I continued the irritation, and it moved forward its right hind leg and passed its toes distinctly over the part. It performed a similar movement with its left hind foot, on its left fore leg being irritated. On compressing the toes of its left hind foot, it made strong efforts to be relieved, and threw itself upon its back. On being replaced upon its face, I continued the irritation, with similar results. But the frog always required to rest a little before the irritation was renewed, that the movements might be distinctly manifested.

*Experiment* 5, April 28th, 1858.—I divided the spinal cord of a frog immediately behind the cranium, and removed every portion of the encephalon anterior to this. Respiration instantly ceased.

I then placed the frog, as in the preceding experiment, in a little water in a vessel, and allowed it to remain at rest for a considerable time, till the effects of the operation had subsided.

I touched with the point of a needle the integuments of the right side of its abdomen, and it raised up its right hind leg and scratched the part with its foot. I irritated the integuments of its right dorsal region, and it scratched the part with its right hind foot. I touched with the point of a needle the integuments of its left cervical region immediately below the division of the cord, and retained the needle on the part, and it raised up its left hind foot and pushed away the instrument with force. I placed the point of a needle on the integuments of its right cervical region, and it raised up its right hind leg and pushed away the needle with its foot. I retained the point of the needle on the integuments of its left dorsal region, and it raised up its left hind leg and pushed its foot against the needle. On the needle being removed, it scratched the part with its foot. It was completely insensible to every irritation of the integuments of its head, till we touched the part below the division of the cord, and then it raised up its hind foot and pushed away the needle, and scratched the part with its toes. I have also observed it raise up its hind foot and scratch the part that had been irritated, a short time after the needle had been removed.

On being placed upon its back, it was unable to turn upon its face. When its foot was compressed it struggled to be relieved, and gave the most distinct evidence of sensation and perception of the stimulus.

